# Supplementary material for: Effect of Ultrasound Treatment on Barrier Changes of Polymers before and after Exposure to Food Simulants
Source: Polymers (Basel). 2022 Feb 28;14(5):990. doi: 10.3390/polym14050990 (PMC8912829; doi:10.3390/polym14050990)
Supplement: Supplementary file 1 [file polymers-14-00990-s001.zip › polymers-1590911-supplementary.pdf]

Supplementary Materials

The effect of ultrasound treatment on barrier changes of polymers before and after exposure to food simulants

Authors: Mario Ščetar[1], Davor Daniloski[2, 3*], Mirela Tinjić[1], Mia Kurek[1], Kata Galić[1]

Affiliations:

[1] University of Zagreb, Faculty of Food Technology and Biotechnology Pierottijeva 6, 10000 Zagreb, Croatia

[2] Victoria University, Advanced Food Systems Research Unit, Institute for Sustainable Industries and Liveable Cities and College of Health and Biomedicine, Melbourne VIC 8001, Australia

[3] Teagasc Food Research Centre, Food Chemistry and Technology Department, Moorepark, Fermoy, P61 C996, Cork, Ireland

* Correspondence: davor.daniloski@live.vu.edu.au; Tel.: +61 48 246 1335

Table S1. Samples treatment and coding.

Packaging material	Food simulant	Ultrasound treatment	Sample code
LLDPE			LLDPE (control)
		5 min at 60 ^{0}C	LLDPE 5 (control)
		15 min at 60 ^{0}C	LLDPE 15 (control)
		30 min at 60 ^{0}C	LLDPE 30 (control)
	HAc 3 % (w/v)	5 min at 60 ^{0}C	LLDPE: HAc 5
		15 min at 60 ^{0}C	LLDPE: HAc 15
		30 min at 60 ^{0}C	LLDPE: HAc 30
	EtOH 10 % (v/v)	5 min at 60 ^{0}C	LLDPE: EtOH 5
		15 min at 60 ^{0}C	LLDPE: EtOH 15
		30 min at 60 ^{0}C	LLDPE: EtOH 30
PPAcPVDC			PPAcPVDC (control)
		5 min at 60 ^{0}C	PPAcPVDC 5 (control)

		15 min at 60 ^{0}C	PPAcPVDC15 (control)
		30 min at 60 ^{0}C	PPAcPVDC 30 (control)
	HAc 3 % (w/v)	5 min at 60 ^{0}C	PPAcPVDC: HAc 5
		15 min at 60 ^{0}C	PPAcPVDC: HAc 15
		30 min at 60 ^{0}C	PPAcPVDC: HAc 30
	EtOH 10 % (v/v)	5 min at 60 ^{0}C	PPAcPVDC: EtOH 5
		15 min at 60 ^{0}C	PPAcPVDC: EtOH 15
		30 min at 60 ^{0}C	PPAcPVDC: EtOH 30

Table S2. Activation energy (*Ea*) for permeation (*P*), solubility (*S*) and diffusion (*D*) of tested materials: untreated (control); food simulant (EtOH and HAc) and ultrasound treatment (5 min, 15 min, 30 min).

	**E_a (kJ/mol)**		
**Sample**	***S***	***D***	***P***
LLDPE (control)	-44.28	-2.28	-46.56
LLDPE: EtOH 5	51.94	-57.13	-5.20
LLDPE: EtOH 15	10.77	-10.89	-0.12
LLDPE: EtOH 30	55.09	-49.51	5.58
LLDPE: HAc 5	-40.54	39.79	-0.75
LLDPE: HAc 15	11.45	-16.40	-4.95
LLDPE: HAc 30	64.88	-67.97	-3.09
PPAcPVDC (control)	-85.37	-13.32	-98.69
PPAcPVDC: EtOH 5	-101.22	61.04	-40.17
PPAcPVDC: EtOH 15	-75.49	-60.04	-81.50
PPAcPVDC: EtOH 30	-62.49	50.35	-12.14
PPAcPVDC: HAc 5	-91.85	18.11	-73.74

PPAcPVDC: HAc 15	26.83	-234.53	-20.77
PPAcPVDC: HAc 30	-20.30	-60.63	-80.94

LLDPE - linear low density polyethylene; PPAcPVDC - polypropylene coated with acrylic/poly(vinylidene chloride; control - treatment time equal to 0 min; EtOH - 10% (v/v) ethanol; HAc - 3% (w/v) acetic acid.

Table S3a. Levels of significance (p value) for the thickness and the water vapour permeability of packaging materials (Pack) after the ultrasound (UST) and food simulant (FS) treatments.

Parameters	Thickness	Water vapour permeability
Packaging material	< 0.001 ***	< 0.001 ***
FS	0.004**	< 0.001 ***
UST	< 0.001 ***	< 0.001 ***
Pack·FS·UST	< 0.001 ***	< 0.001 ***

*≤ 0.05 *; ≤ 0.01 **; ≤ 0.001 ****

Table S3b. Levels of significance (p value) for the oxygen permeability parameters of packaging materials (Pack) after the ultrasound (UST) and food simulant (FS) treatments.

Parameters	Gas permeance (q)	ln S	ln D	ln P
Packaging material	< 0.001 ***	< 0.001 ***	< 0.001 ***	< 0.001 ***
Temperature	< 0.001 ***	< 0.001 ***	< 0.001 ***	< 0.001 ***
FS	< 0.001 ***	0.131	0.026*	0.454
UST	< 0.001 ***	< 0.001 ***	0.734	< 0.001 ***
Pack·Temperature·FS·UST	< 0.001 ***	< 0.001 ***	< 0.001 ***	< 0.001 ***

*≤ 0.05 *; ≤ 0.01 **; ≤ 0.001 ***.*

Table S3c. Levels of significance (p value) for the overall migration (OM) of packaging materials (Pack) after ultrasound (UST) and food simulant (FS) treatments.

Parameters	Overall migration
Packaging material	< 0.001 ***
FS	< 0.001 ***
UST	< 0.001 ***
Pack·FS·UST	≤ 0.001 ***

*≤ 0.05 *; ≤ 0.01 **; ≤ 0.001 ***.*
